# Peptide–nanoparticle conjugates: a next generation of diagnostic and therapeutic platforms?

**DOI:** 10.1186/s40580-018-0170-1

**Published:** 2018-12-12

**Authors:** Woo-jin Jeong, Jiyoon Bu, Luke J. Kubiatowicz, Stephanie S. Chen, YoungSoo Kim, Seungpyo Hong

**Affiliations:** 1 Pharmaceutical Sciences Division, School of Pharmacy, The University of Wisconsin-Madison, 777 Highland Ave., Madison, WI 53705 USA; 2 Integrated Science and Engineering Division, Department of Pharmacy, Yonsei Institute of Pharmaceutical Sciences, Yonsei University, Incheon, 21983 Republic of Korea; 3 Yonsei Frontier Lab, Department of Pharmacy, Yonsei University, Seoul, 03722 Republic of Korea

**Keywords:** Peptide–nanoparticle conjugates, Drug delivery, Protein interaction inhibitor, Molecular imaging, Liquid biopsy

## Abstract

Peptide–nanoparticle conjugates (PNCs) have recently emerged as a versatile tool for biomedical applications. Synergism between the two promising classes of materials allows enhanced control over their biological behaviors, overcoming intrinsic limitations of the individual materials. Over the past decades, a myriad of PNCs has been developed for various applications, such as drug delivery, inhibition of pathogenic biomolecular interactions, molecular imaging, and liquid biopsy. This paper provides a comprehensive overview of existing technologies that have been recently developed in the broad field of PNCs, offering a guideline especially for investigators who are new to this field.

## Introduction

**Fig. 1:**
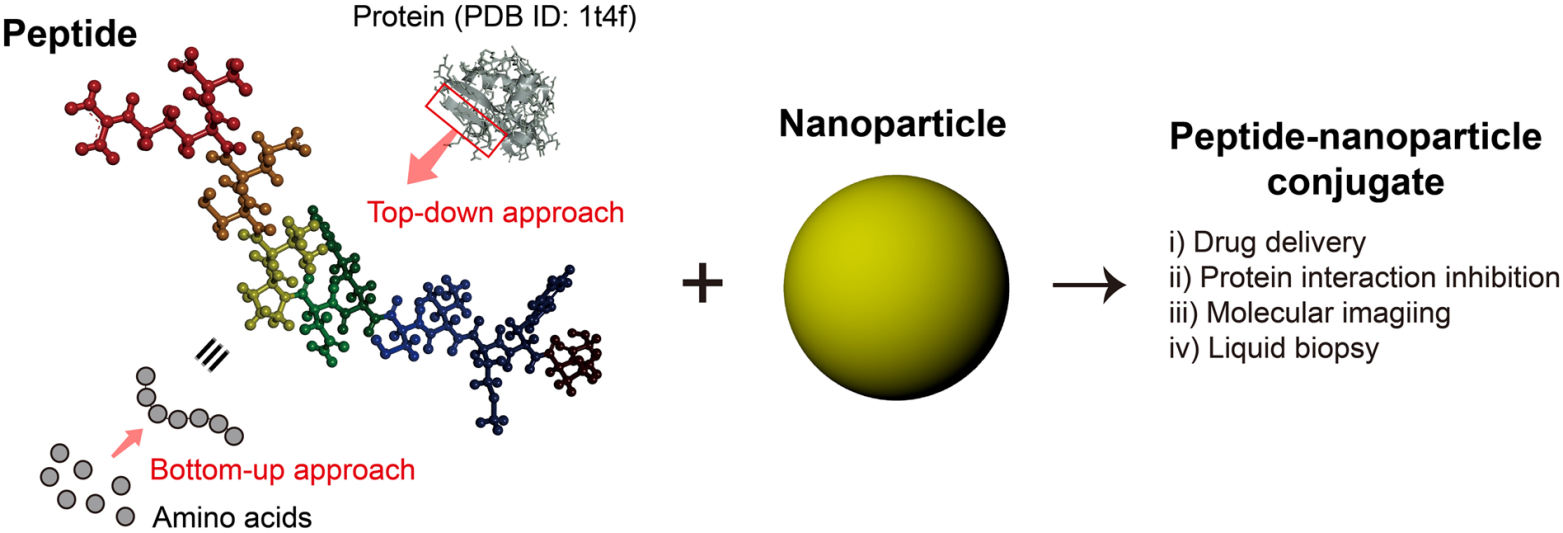
Discovery of artificial bioactive peptides and their conjugation with nanoparticles for biomedical applications

Peptides have attracted a great deal of interest in biomedical fields as a novel material that can both exhibit protein functionalities and possess a high degree of modularity in molecular design. Current strategies for the discovery of artificial bioactive peptides can be broadly divided into two categories (Fig. 1): (i) the construction and screening of peptide libraries from random amino acid compositions within a certain macromolecular topology (peptide library screening, bottom-up approach) and (ii) the isolation of bioactive sequences from natural proteins based on their three-dimensional (3D) structures (structure-based design, top-down approach) [1–4]. Peptide library screening enables the facile development of effective binders against a wide range of target molecules (e.g. small molecular compounds, peptides, DNAs, RNAs, cells, and inorganic materials). The top-down method, on the other hand, has an advantage over the bottom-up approach as peptide sequences aiming to a specific binding site on biomacromolecules can be discovered based on their structural properties.

Over the past decades, a large number of studies have demonstrated the utility of artificial bioactive peptides and some of these products have been successfully commercialized. Specifically, 28 noninsulin peptide drugs have been approved worldwide during the last two decades with several being highly competitive in the market [5]. In addition, over 150 peptide drugs are in active clinical development, demonstrating highly promising results for ultimate translation [6]. Despite the recent strides, most peptides have yet been widely utilized due to: (i) their lower target binding affinity and selectivity than proteins; (ii) vulnerability to protease digestion in biological environments [7]; (iii) short circulating half-lives resulting in the requirement for frequent administrations to sustain their efficacy [8]; and (iv) inability to maintain innate folding structures when isolated from protein contexts, which significantly limits their function [9].

Many researchers have found that the incorporation of peptides with non-biological materials (e.g. small molecular compounds, metal chelates, polymers, and hydrogels) is a promising approach to addressing the intrinsic drawbacks of the peptides [10, 11]. Particularly, nanoparticles (NPs) have shown their potential to serve as conjugate scaffolds that not only improve the functionality of peptides but also implement abiotic characteristics, often resulting in synergistic effects (Sect. 2). As a result, peptide–NP conjugates (PNCs) have been considered a promising platform for a variety of biomedical uses. This review therefore focuses on PNCs, highlighting the recent progress in the PNCs-based technologies and their uses in diagnostic, imaging, and therapeutic applications. The advantages of employing PNCs will be briefly discussed first (Sect. 2), followed by description of examples of their successful applications to biomedical areas, including targeted drug delivery (Sect. 3), pathogenic protein interaction inhibition (Sect. 4), highly sensitive molecular imaging (Sect. 5), and liquid biopsy (Sect. 6). Finally, we will provide a perspective on the research applications that have been rapidly developed but still suffer from several challenges for clinical translation.

## The peptide–NP conjugation

Nanomaterials (tens to a few hundreds of nanometers in size) possess novel physico-chemical properties distinct from those of conventional bulk materials. Their ultra-small size and high surface-area-to-volume ratio are advantageous in the development of engineered materials that can uniquely interact with a variety of nano- and micro-sized biomaterials [12]. The most straightforward approach to fabricate peptides-based nanostructures is self-assembly [13, 14]. However, the spontaneity in the thermodynamic process does not allow the construction of nano-scale constructs having precisely regulated shape, size, and compositions. In contrast, peptide–NP conjugation offers enhanced control over the structural properties of nanostructures, allowing facile modification to overall shape, dimension, and size of the conjugates through engineering NP scaffolds tailored for intended applications.

**Fig. 2:**
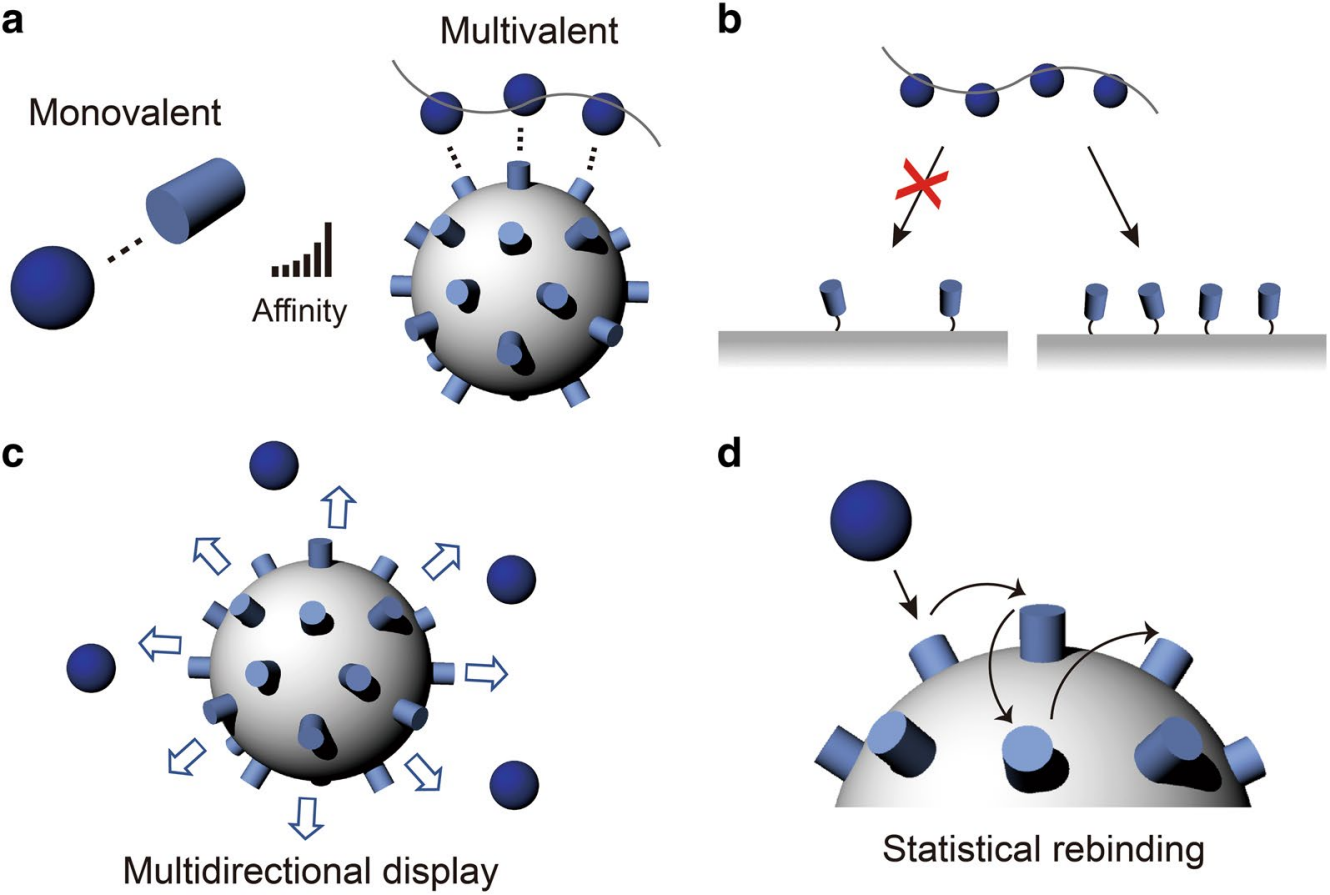
**a** Comparison between monovalent- and multivalent interactions. **b** Selectivity in multivalent interactions. **c** Multidirectional ligand display and **d** statistical rebinding on a multivalent object

Another important aspect that the PNCs can provide is multivalency. Most interactions in biological systems are based on non-covalent interactions such as hydrogen bonds, ionic bonds, van der Waals forces, π–π stacking forces, and hydrophobic interactions. Although the individual bindings are relatively weak, their co-operative action enables strong binding kinetics (typically due to substantial decrease in dissociation kinetics through the multivalent binding effect) based on the principle that the collective binding strength depends exponentially on to the number of individual binding pairs (Fig. 2a) [15–17]. In addition to the enhanced binding strength, multivalent interactions also provide improved selectivity by exploiting the density of interaction modules on a surface to recognize target polyvalent surfaces (Fig. 2b) [18].

The presence of multiple binding sites plays a role in allowing the strong multivalent bindings and in increasing statistical opportunities for multiple monovalent binding events to occur. As depicted in Fig. 2c, the exposure of peptides in multiple directions results in greater opportunities to encounter binding partners [19]. During the dissociation process post binding, peptides on NP scaffolds express many re-binding sites, which can increase the retention time of target materials on the surface, known as the statistical re-binding mechanism (Fig. 2d) [20]. Furthermore, co-conjugation with different types of peptides and/or other biological/non-biological materials offers additional functionalities for the hybrid materials, such as immune response evasion [21], theranostics [22], stimulus-responsive property [23], and multi-target directed treatment with a single material [24]. Consequently, displayed on a nanostructure surface, peptides can potentially compete with or outperform natural proteins despite their low individual affinity and selectivity [25, 26].

The non-biological characteristics of NPs introduce novel properties and functions that are otherwise not obtained to their PNCs. For instance, NPs absorbing and emitting near infrared (NIR, 700–1100 nm) light have been actively utilized in in vivo imaging due to the advantages of deep imaging depth and high spatial resolution [27]. Some NPs produce reactive oxygen species (ROS) upon receiving the light energy, which can oxidize biomacromolecules and subsequently induce cell ablation (photodynamic therapy) [28]. In addition, the absorbed light energy can be converted to heat and sound energy using photothermal and photoacoustic effects of NPs, providing a non-invasive treatment option for diseases like cancer [29, 30]. Magnetic nanoparticles (MNPs) are another promising class enabling the remote and active treatment of diseases. Responding to external magnetic stimuli, MNPs can be selectively accumulated at a target site in biological systems and release guest molecules in a dosage-controlled fashion [31, 32]. Several in vitro studies have shown that MNPs, displaying multiple binding ligands, effectively discriminate target biomaterials from a mixture solution [33]. Furthermore, upon exposure to the magnetic field, the arrangement of MNPs on a surface can be controlled in various ways, resulting in the use of the MNPs for the development of novel cell culture scaffold [34].

## Targeted drug delivery

Selective delivery of pharmaceutical agents to target sites in the body remains a major challenge. Peptides have recently emerged as a powerful arsenal that may provide modular selectivity to drug delivery systems, warranting enhanced performance for the potential treatment of many serious health problems, such as cancer and brain diseases [35, 36]. Peptides specifically interact with different types of biological systems, allowing them to be applied in a multitude of scenarios for effective results [37]. However, the short in vivo half-life time and sub-optimal biodistribution and pharmacokinetics of peptides have hindered their widespread applications in drug delivery [38].

A simple approach to overcoming the problems of the current peptide-based delivery system is to combine them with NPs. Upon functionalization with peptides as targeting agents, NPs can be engineered to selectively deliver the drugs to the target tissue, in addition to their capability to encapsulate and protect therapeutic agents, increasing the plasma circulation time. As a result, researchers have conjugated different targeting peptides on different types of NPs to provide more efficient and adaptable drug delivery systems (Table 1). One of the specific applications of peptide-mediated targeting is the delivery of cargo to the nucleus of cells. Delivery to the nucleus is particularly difficult due to the many barriers that must be overcome once inside the cell, let alone targeting to the correct cell in the first place. Most particles enter the cell via endocytosis and are thus encapsulated in large vesicles, headed towards a lysosome for degradation. They must have some means of endosomal escape to avoid being destroyed before they can reach the nucleus [39]. Once this is achieved, the particle must bypass the protections afforded to the nucleus. The nucleus is protected by a double phospholipid membrane, accessible mainly through nuclear pore complexes (NPCs), which have varied diameters ranging from approximately 20 to 150 nm [40]. Not only must the particle be small enough to make it through, it must also have a corresponding nuclear localization signal (NLS), which acts a key card to allow access through the NPC. Pan et al. developed a solution to these problems in vitro by utilizing mesoporous silica NPs conjugated with TAT peptide for the delivery of doxorubicin (DOX) to the nucleus of HeLa cells [41]. Their results show that particles smaller than 50 nm were able to achieve TAT peptide-mediated nuclear uptake and continuous release of DOX into the nucleus over a 24-h incubation period. A different approach was taken by Tkachenko et al., who employed a multi-peptide conjugated gold NP (AuNP)-based system for this purpose [42]. They reported that the use of two short peptides that are introduced for cellular endocytosis and for nuclear targeting of the particle is more effective than attempting to use a single lengthy sequence. The 25 nm AuNP was able to enter the nucleus in 80% of HepG2 cells when incubated for 2 h at 37 °C. Li et al. similarly utilized a 13 nm AuNP-based system conjugated with an NLS peptide although their aim was to deliver siRNA for gene silencing [43]. They reported that their complex was able to successfully hinder TK1 protein and TK1 mRNA prevalence in vitro and reduce tumor growth by 250% when compared to a control for an in vivo mouse model.

**Table 1:** Peptide–nanoparticle conjugates for efficient drug delivery

Application	Peptide		Nanoparticle (NP)		Therapeutic agents	In vitro study		In vivo study		Refs.
	Name	Target	Type	Complex size (nm)		Model	Efficacy	Model	Efficacy	
Nuclear-target drug delivery	TAT	Target importin alpha and beta for intranuclear translocalization	Mesoporous Silica	25, 50	Doxorubicin	MTT Assay for DOX-Carrier Cytotoxicity	Hela cell viability: ~ 30%	N/A	N/A	[41]
	Adenoviral NLS	Interact with nuclear pore complex for nuclear uptake	BSA-coated AuNP	25	Preliminary study (N/A)	LDH colorimetric toxicity assay for Carrier Cytotoxicity	HepG2 cell viability: < 5% decrease compared to control	N/A	N/A	[42]
	Adenoviral RME	For receptor mediated endocytosis into the cell								
	Adenoviral NLS	Targets nuclear pore complex for NP entrance into nucleus	AuNP	13	SiRNA	MCF-7 (Breast), HeLa (Cervix), HepG2 (Liver) cancer cells	TK1 mRNA expression decreased 10%	MCF7 tumor-bearing mice	Inhibited tumor growth. ~ 2.5× lower weight than control	[43]
Transdermal drug delivery	TAT	Assists with membrane disruption and cellular uptake	AuNP	200	pDNA	Nude mouse skin	Past epidermis and within dermal layer	N/A	N/A	[44]
						Transfection of B16F10 Cells	1.71 * 10^7^ RLU/mg (significantly higher)			
	TD	Targets the Na^+^/K^+^-ATPase beta-subunit of the stratum corneum for enhanced skin permeability	Liposome	105	Vemurafenib	Franz diffusion cell system	~ 60 µg Vem quantity in receptor after 24 h. (significantly higher)	BALB/c nude mice	Significant antitumor efficacy	[46]
	TAT	Arginine groups in TAT bind stratum corneum and assist NP movement into epidermal layers	Nano lipid crystal NPs	180	Celecoxib	Hairless rat skin permeation using Franz diffusion cells	Threefold higher conc. in stratum corneum. Highest epidermal concentration (90 µg/g of skin). Max depth 120 µm	N/A	N/A	[45]
Blood brain barrier drug delivery	G23	Targets gangliosides GM1 and GT1b for the mediated transport of NPs across the BBB	Polymersome	165	Preliminary study (N/A)	hCMEC/D3 cells on transwell filters	~ 30% transcytotic capacity (4 times increase over nontargeted)	BALB/c nude mice	Significant accumulation in brain parenchyma. Also, accumulation in cortex, striatum, midbrain, pons and cerebellum	[48]
	LNP	Cell penetrating peptide for cellular uptake	DGL-PEG	90	pDNA	BCEC cells in well plates	P_app_ achieved 92.43 * 10^−6^ cm/s and ~ 275 pmol total transport (both significantly higher)	Nude orthotopic glioma-bearing mice	Increased median survival time and statistically significant survival prolongation	[49]

Another interesting application for NPCs involves transdermal delivery for the treatment of melanoma. The main barrier preventing delivery for this application is the stratum corneum, the outermost layer of skin. Niu et al. designed a AuNP-based system that employed conjugated TAT peptides for the delivery of plasmid DNA (pDNA) [44]. Their results confirmed that TAT peptides boost skin infiltration and gene transfection of NPs for an effective topological delivery system. Patlolla et al. also took advantage of the skin permeation capabilities of TAT peptides by conjugating them to nano lipid crystal NPs (NLCNs) with 180 nm in size [45]. They reported that their complexes penetrated up to 120 µm into an in vitro rat skin, with higher concentrations of particles accumulated in both the stratum corneum and epidermal layers, when compared to other complexes tested. Zou et al. tackled this problem in a different manner, choosing to use a liposome NP conjugated with TD peptide for the delivery of Vemurafenib [46]. Their data indicates TD peptides’ capacity to open the paracellular pathways of the stratum corneum for transdermal delivery to melanoma.

Peptides have been also found to be useful for assisting NPs across other physiological barriers, including the blood brain barrier (BBB) that represents a major hurdle for effective delivery of pharmaceutical agents to the brain. The BBB acts as a shield surrounding blood vessels with access to the brain; its main purpose is to prevent non-essential substances from reaching the delicate system behind it [47]. Researchers have been using peptides to help NPs transport across the BBB. For instance, Georgieva et al. used G23 peptide-conjugated polymersomes for both in vitro and in vivo delivery of drugs across the BBB [48]. The 165 nm NPs utilized G23 peptide to target ganglioside GM1 and GT1b receptors expressed on hCMEC/D3 cells (human BBB model), enabling four times greater transcytotic capacity over polymersomes without G23 peptide. Another group, Yao et al., reported their use of a dendrigraft poly-l-lysines (DGL) NP conjugated with poly(ethylene glycol) (PEG) and a LIM Kinase 2 derived cell-penetrating peptide (LNP) for the delivery of pDNA across the BBB [49]. Their novel system took advantage of LNP that facilitates cellular uptake by peripheral cells present in the BBB.

The PNC-based approaches have demonstrated a number of successful examples that have achieved efficient targeting to diseased cells and permeation across physiological barriers. However, there are many challenges that need to be overcome for ultimate translation of this approach, such as immunogenicity, long-term toxicity, and off-targeting potential. Upon addressing those concerns, it is foreseeable that the PNC approach will provide a powerful method for efficient drug delivery with high therapeutic index.

## Pathogenic Protein Interaction Inhibition

Drugging the ‘undruggable’ targets is one of the key challenges in pharmacological studies [50]. Approximately 80% of proteins that involved in human diseases lack binding sites for small molecule ligands [51]. One potential strategy to address this issue is to implement protein-based pharmaceuticals. However, low thermal stability and difficulty in preparation of such proteins have hindered their widespread application [52].

**Fig. 3:**
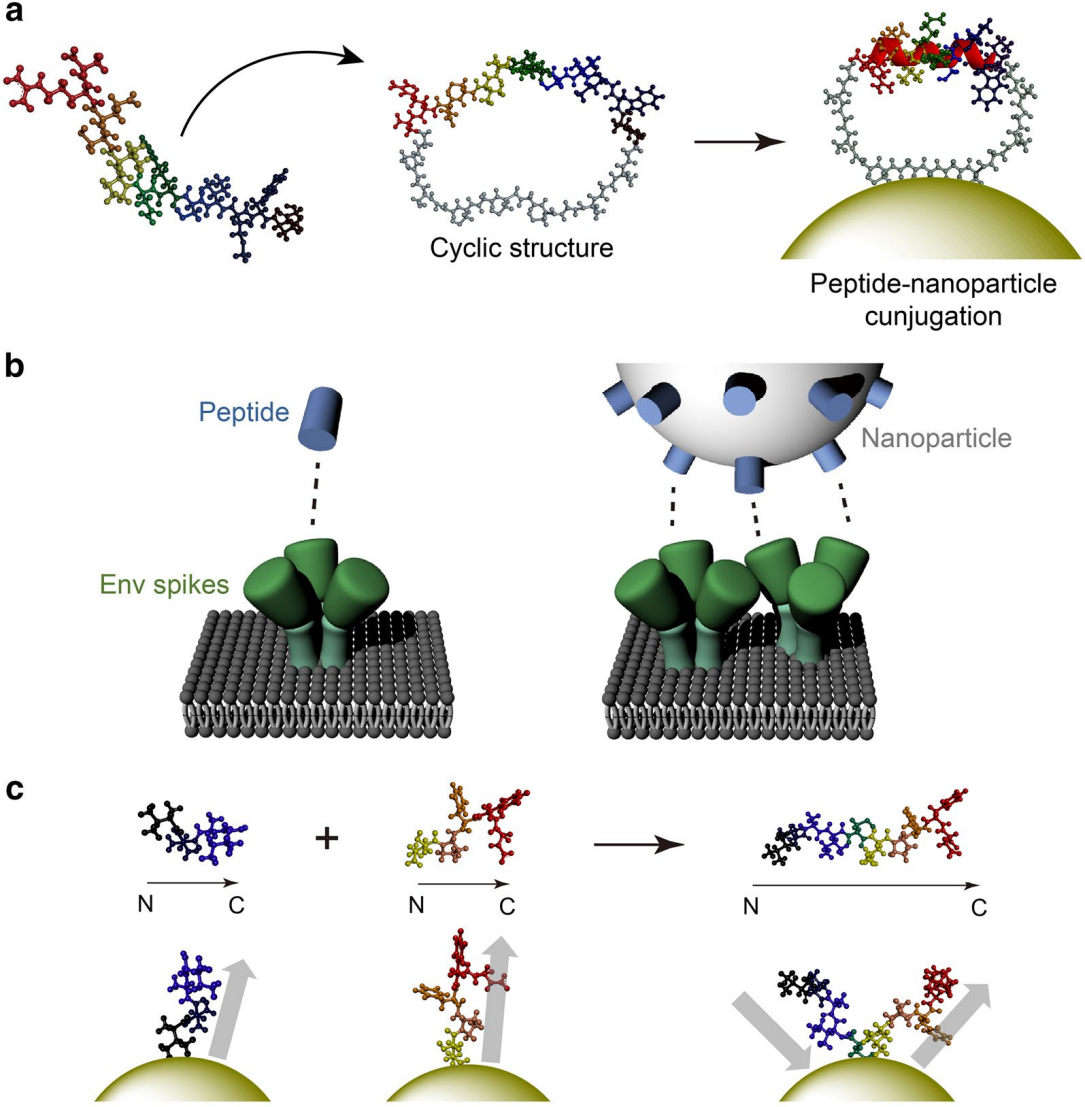
**a** Molecular models depicting gold nanoparticle binding-induced stabilization of α-helical structure. **b** Interactions of free peptides and peptide–nanoparticle conjugates with HIV-1 spike proteins. **c** Peptide hybrid-functionalized gold nanoparticles inhibiting amyloid-β aggregation

PNCs provide a new insight to tackle these formidable challenges. For instance, the Lim group demonstrated inorganic NPs that serve as a scaffold for stabilizing peptide folding structures, which can eventually enhance both target affinity and selectivity [53]. Figure 3a illustrates α-helical structure stabilized by reduced conformational entropy cost achieved through the use of cyclic peptides and interaction with inorganic surface [54]. Based on this principle, bioactive α-helical p53 peptides stabilized on AuNP surfaces effectively recognized their target protein, MDM2, which is known to suppress the p53-mediated apoptotic pathway. The therapeutic potential of the cyclic peptide–nanomaterial conjugate system was also demonstrated by inhibiting the α-helix-mediated interaction between Rev protein and Rev response element (RRE) RNA, which regulates HIV-1 gene expression [24, 55].

The multivalent property of PNCs is a powerful tool for controlling polyvalent macromolecular associations that frequently occur in nature. Chaiken et al. reported that AuNP–peptide triazole conjugates inactivates HIV-1 by disrupting the interactions between host receptor proteins and trimeric envelope glycoprotein (Env) spikes of the virus [56, 57]. As AuNP diameter and peptide valency increase, the antiviral potency of the PNCs is greatly enhanced. This implies that a sufficient quantity of peptide triazoles over a large area is required for effective interaction with the multiple spikes on the viral surface (Fig. 3b). Protein-misfolding diseases including Alzheimer’s disease (AD) are also difficult to target with conventional therapeutics [58]. Xiong et al. decorated AuNPs with peptides including two inhibitory peptide sequences for Aβ aggregation, VVIA and LPFFD, in order to develop a multivalent inhibitor for the aggregation of amyloid-β (Αβ) proteins [59–61]. The two peptide sequences were conjugated onto the AuNP surfaces and ordered/oriented in optimal conformation to effectively inhibit Aβ aggregation. Utilizing the two different peptides on a single NP was highly synergistic, preventing Aβ aggregation more strongly with less cytotoxicity, compared to the free peptides (Fig. 3c).

In some applications, PNC functionality can be significantly improved by precisely controlling the peptide valency. NPs that are covered with peptides at a higher density typically exhibit increased binding affinity [62]; however, precisely engineered binding modules that have a specific spacing or certain ligand density have been shown to further enhance the interaction with target molecules in a controlled manner [63, 64]. One approach to controlling the ligand valency is to use dendrimers. Dendrimers are hyper-branched polymers that have precisely controlled size, surface property, composition, and density of functional groups through relatively simple chemical reactions [65, 66]. In a recent study, Lauster et al. showed that polyglycerol dendrimers decorated with peptides targeting hemagglutinin (HA) can inhibit the infection of influenza A virus (IAV) [25], which uses multiple HAs for enhanced binding to the host cell surface [67]. Interestingly, despite the improved antiviral activity of the PNC utilizing the multivalent binding of the HA targeting peptides, the inhibitory capacity was not proportionally increased with an increase of the peptide density. Instead, higher valency reduced the inhibitory activity when it exceeded a certain threshold, indicating that optimization of the surface engineering is required.

Another advantage of PNCs is that they can utilize multiple therapeutic pathways by incorporating different types of molecules in a single nanoformulation system [68]. Recently, Blancafort et al. conjugated poly(glycidyl methacrylate) NPs with peptides targeting Engrailed 1 (EN1), an undruggable transcription factor associated with cell proliferation, metastasis, and chemoresistance of basal-like breast cancer [69]. An anticancer agent, docetaxel (DTX), was encapsulated in the internal void of this NP. Both in vitro and in vivo studies revealed that the combination of peptidic- and chemotherapeutic agents via PNC induced more apoptosis on cancer cells, compared with using either DTX or EN1 peptide alone. Alternatively, Jeong et al. demonstrated that conjugation of different types of peptides onto a nanomaterial is an effective way to maximize therapeutic effect [24]. In their study, two different peptides were conjugated on carbon nanotubes to inhibit Rev/RRE RNA and Rev/CRM1 interactions, resulting in 150-fold enhanced HIV-1 inhibition, compared to leptomycin B, a commonly used HIV-1 inhibitor [70].

As described above, peptides have shown great potential to overcome their intrinsic limitations when conjugated onto NP surfaces. It has been reported that PNCs could outperform single peptides and even proteins, showing higher binding affinity, selectivity, and, in turn, therapeutic effect. This PNC approach has been also proven useful in other applications, such as molecular imaging and diagnostic/prognostic applications, including liquid biopsy, which will be discussed in the following sections.

## Molecular imaging

Molecular imaging provides visual information on biological processes at high resolution [71]. It enables detection of pathological cells and tissue, helping both pre-clinical researchers and clinicians understand the status of diseases in terms of their progression and responsiveness to treatments [72]. Recent advances in nanobiotechnology further accelerated the development of molecular imaging by enhancing the targeting efficiency of imaging probes [73]. Among many agents that have been used to provide selectivity, peptides have been successfully employed as novel nanoprobes due to their long-term stability, target-specificity, and rapid clearance from the blood stream [37, 74]. The modular nature of such peptides allows to be integrated with a variety imaging modalities, resulting in remarkable outcomes in animal models and preclinical studies.

Despite their advantages, peptides often suffer from weak binding affinity, metabolic instability, and fast renal clearance due to their small size [75]. These problems can be addressed by conjugating them to NPs, which have been frequently utilized to improve the pharmacokinetics of the targeting peptides [37]. NPs can be selected to fit a variety of target sites and imaging modalities, making them an ideal delivery platform. A major advantage that peptide/NP complexes provide is their ability to enhance the target-to-background signal. This could be accomplished by conjugation of multiple imaging probes onto a NP’s surface or by an increased surface density of specific peptides [76]. Conjugation of different types of peptides, along with therapeutic agents, would enable PNCs to be applied for multitarget-directed nanotherapeutics. This section summarizes recent advances achieved through the use of PNCs as imaging nanoprobes for different applications, including near-infrared (NIR) fluorescence imaging, computed tomography (CT), positron emission tomography (PET), magnetic resonance imaging (MRI), and multi-modal imaging (Table 2).

**Table 2:** Peptide-nanoparticle conjugates for molecular imaging nanoprobes

Imaging		Peptide		Nanoparticle (NP)		Animal studies	Results	Refs.
Modality	Probe	Name	Target or role	Type	Size			
NIR	FITC	DEVD peptide sequence	Cleave caspase-3	Biotinylated NP, Acetyl-Asp-Glu-Val-Asp-Cys(StBu)-Lys(Biotin)-CBT	100–300 nm	N/A	Twofold enhanced (fluorescent intensity, vs. SA-FITC)	[80]
	Zn^2+^ coordinated cyclic peptide NP (f-PNP)	RGD	Targets α_v_β_3_ Integrin	Fluorescent cyclic peptide NP (f-PNP, self-assembled)	28 nm	Xenografted EC mouse model	Highly photostable and narrow emission spectrum	[81, 82]
	Small-molecule NIR-II organic dye	RM26 peptide	Targets gastrin-releasing peptide receptor	DSPE-mPEG NP	60 nm	U87MG (glioblastoma) tumor bearing mouse model	Highly sensitive and specific to GRPR	[83]
CT	AuNP	RGD	Targets α_v_β_3_ integrin	Dendrimer-entrapped gold nanoparticles (Au DENPs)	4.0 nm (Au core)	N/A	Enhanced X-ray attenuation compared to Omnipaque	[84]
	AuNP + IR780 (Fluor)	Angiopep-2	Targets glioma	DTX-loaded PLGA@Au NP	180 nm	U87MG (glioblastoma) tumor bearing mice	4 h (Whitening effect AT the target site)	[85]
	AuNP + Cy5.5 (Fluor)	Fibrin-targeting peptide and Thrombin-activatable fluorescent peptide	Targets fibrin and Cleave thrombin	Glycol-chitosan-coated AuNP (GC-AuNP) and SiO_2_@AuNP	127 nm (Pep-GC-AuNP) and 39.8 nm (Pep-SiO_2_@AuNP)	C57Bl/6 mouse model	Remained at the target site for up to 3 weeks	[86, 87]
PET	^18^F	CK and CLPFFD peptides	Targets β-amyloid fiber	AuNP	12 nm (hybrids)	Sprague–Dawley rat model	NPs were trapped by reticuloendothelial system (RES)	[89, 90]
	^64^Cu	RGD	Targets α_v_β_3_ integrin	Au-tripods	10–15 nm	U87MG (glioblastoma) tumor bearing mice	Threefold enhanced (PAI contrast, vs. blocking group)	[91]
	^125^I ^76^Br	RGD	Targets α_v_β_3_ integrin	PEO dendrimer	12 nm	Unilateral hindlimb ischemia-induced mice	50-fold enhanced (affinity, vs. free peptide) > twofold enhanced (ischemic to nonischemic hindlimb ratio, vs. nontargeted NP)	[92]
MRI	Iron oxide	RGD	Targets α_v_β_3_ integrin	Iron oxide NP	< 10 nm (NP) 8.4 nm (Hybrid)	U87MG (glioblastoma) tumor bearing mice	42% (tumor MR signal intensity reduction, 15% for free peptide)	[94]
	Iron oxide	RGD	Targets α_v_β_3_ integrin	Superparamagnetic polymeric micelles (SPPM): SPIO NPs inside the core of a PEG-PLA co-polymer micelle	9.9 nm (SPIO) 50–75 nm (SPPM)	A549 (lung), MDA-MB-231 (breast), U87MG (Glioblastoma) tumor bearing mice	10^−12^ mol/L (detection limit)	[95, 96]
	Iron oxide	CREKA	Targets fibrin	Amino dextran-coated SPIO	50 nm	Mouse model	NPs accumulates in tumor vessel → self-amplifying tumor homing	[97]
Multi-modal	Hollow Au nanosphere (HAuNS, CT) ^64^Cu (PET)	RGD	Targets α_v_β_3_ integrin	HAuNS	44.7 nm	VX2 tumor-bearing rabbit model	0.20% (tumor uptake, vs. 0.099% for non-RGD NP)	[98]
	Cy5 (Fluor) Gd (MRI)	Activatable cell penetrating peptides (ACPPs)	Targets active MMP-2 and -9	G5 PAMAM dendrimer	4.6 nm	HT-1080 (fibrosarcoma) tumor-harboring mice	4- to 15-fold enhanced (NP uptake, vs. unconjugated peptides)	[99]

NIR fluorescence imaging utilizes imaging agents with emission spectra in between 700 and 1100 nm [77]. NIR light penetrates deeper into the tissue than the visible light, allowing enhanced tissue imaging [77, 78]. Recently, PNCs have been applied for NIR imaging, allowing sensitive detection of abnormal tissue with high specificity [79, 80]. Fan et al. developed fluorescent NPs consisting of cyclic peptides that were co-assembled with Zn^2+^ ions to generate strong NIR fluorescent signals [81, 82]. This imaging agent was further modified with α_v_β_3_ integrin-specific RGD peptides to selectively image the tumor site. This tumor-specific imaging agent was highly photostable and showed a narrow emission spectrum, resulting in clear NIR fluorescent signals from the target tissue. Benzo-bis(1,2,5-thiadiazole) fluorophores have also been exploited for NIR imaging [83]. The fluorophores were coupled with peptides that are specific to gastrin-releasing peptide receptor (GRPR). Both in vitro and in vivo data demonstrated that these conjugates effectively accumulate at a target tissue with high target-to-background signals.

CT scans rely on multiple X-ray beams, generating cross-sectional images of bones, blood vessels, and soft tissues inside the body. AuNPs are one of the most commonly used imaging agents for CT scans, due to their high X-ray attenuation capability. Their biocompatibility, stability, and versatility enable AuNPs to be utilized in a wide range of applications [37]. Conjugated with peptides, AuNPs have been employed as selective CT contrast agents. Zhu et al. decorated AuNP-entrapped dendrimers (AuDENPs) with RGD peptides and applied these nanoprobes for CT tumor imaging [84]. X-ray attenuation of AuDENPs was superior to that of Omnipaque™, a commonly used CT imaging agent. Recently, Hao et al. developed a core–shell structured NP composed of poly(lactic-*co*-glycolic acid) (PLGA)-AuNP [85]. This NP was conjugated with Angiopep-2, a glioma targeting peptide, that enhanced selective cellular uptake of PLGA–AuNPs, resulting in increased tumor recognition and improved resolution of CT images. The PNC-based approach has been also applied for visualizing cerebral cerebrovascular thrombi. Glycol-chitosan-coated AuNPs (GC-AuNPs) were incorporated with fibrin-specific peptides for direct CT-based imaging of cerebrovascular thrombi [86, 87]. This novel imaging agent selectively accumulated to the target site and were retained in the site for a longer period of time (up to 3 weeks), compared to GC-AuNPs without the peptide. The improved selectivity and longer imaging capability would allow this system to detect short-term recurrence without additional injections.

PET is accepted as an excellent, non-destructive imaging tool for screening various diseases. Incorporation of target-specific peptides and positron emitters to NPs enables highly specific detection of abnormal tissues [88]. CLPFFD peptides targeting β-Amyloid fibers were conjugated with ^18^F-labeled AuNPs to image the biodistribution of the targeted NPs [89, 90]. In another study, Cheng et al. modified the surface of Au-tripod with RGD peptides and ^64^Cu (^64^Cu-RGD-Au-tripods) to provide dual functionalities of integrin-specific targeting and PET imaging, respectively [91]. The administration of this novel PNCs in tumor bearing mouse models led to a threefold enhancement in photoacoustic imaging (PAI) contrasts compared to the PNCs co-injected with free RGD peptides. The PET images also revealed that approximately 8% injected dose of the NPs accumulated and remained in the target site, even after 24 h post injection. Biodegradable dendritic PET nanoprobes with RGD peptides have also demonstrated great potential for screening angiogenesis [92]. The binding affinity of the nanoprobe–peptide conjugates was 50 times higher than that of monovalent peptides due to multivalent interactions. The study was extended to both in vitro and in vivo PET imaging after labelling the conjugates with ^125^I and ^76^Br, respectively, demonstrating that the targeted nanoprobes exhibit enhanced cellular uptake, compared to non-targeted counterparts.

MRI generates high-resolution three-dimensional images of organs and tissues using radio waves and magnetic fields [93]. Magnetic NPs (MNPs) have been utilized as MR contrast agents, and their complexation with targeting peptides has been used to image specific organs. Xie et al. showed that MNPs conjugated with RGD peptides selectively targeted cells that highly expressed α_v_β_3_ integrin [94]. Their in vivo MRI results confirmed that the selectivity of the conjugates were maintained in U87MG tumor-bearing mice. MNPs have been also conjugated with polymers for enhanced targeting and prolonged detection. For example, RGD peptide-conjugated superparamagnetic polymeric micelle (SPPM) nanoprobes have been used for selective detection of integrin overexpressing cells [95, 96]. These nanoprobes were found to selectively accumulate into the tumor site, resulting in detection of the MRI signals from the brain, lung, and breast tumor bearing mice within 5 min post injection. Alternatively, Simberg et al. conjugated the fibrin-specific, tumor-homing CREKA peptide to amino dextran-coated supraparamagentic iron oxides (SPIOs) for targeted imaging and therapy [97]. These conjugates accumulated at the tumor site, self-amplified, and enabled effective MR imaging with high selectivity.

Although PNCs have significantly improved the image quality of various modalities, more accurate and higher resolution imaging is necessary for early diagnosis and effective treatment. Multimodal contrast agents have been recently developed to help researchers and clinicians visualize two or more imaging modalities simultaneously. For example, ^64^Cu-labelled hollow gold nanospheres (^64^Cu-HAuNSs) were engineered to integrate CT and MRI capabilities into a single NP system [98]. The RGD peptides were then immobilized onto the surface of ^64^Cu-HAuNSs to achieve selective targeting and increased cellular uptake of the NPs, resulting in highly selective dual imaging agent for both CT and MRI. Another common strategy involves the combination of fluorescence and MR imaging. Dendritic hybrid NPs, functionalized with activatable cell penetrating peptides (ACPPs) on their branches, were labeled with both Cy5 and Gd for fluorescence and MRI, respectively [99]. ACPPs enhanced cellular uptake of the NPs by up to 15-fold, demonstrating the potential of this system to be used for sensitive detection of tumors via NIR and MR integrated imaging.

The examples described above clearly indicate the potential of PNCs as imaging contrast agents for a variety of modalities. Various peptides have been successfully employed for site-specific targeting in the field of biomedical imaging, which could be further improved using multivalent interaction of PNCs, providing high quality images of specific tissues and organs. Although their potential toxicity and biological instability need to be addressed for the successful clinical translation, PNC-based molecular imaging holds great promise to innovate current diagnostic and therapeutic platforms.

## Liquid biopsy

Liquid biopsy is of high potential significance as a novel tool for diagnosis and prognosis of human diseases [100]. It refers to any techniques that examine, detect, and analyze disease biomarkers in bodily fluids, most notably blood [101]. Given its less invasive nature unlike conventional solid biopsy, liquid biopsy would substantially decrease the chance to cause complications while increasing patients’ compliance, allowing more frequent screening, early detection capability, and more accurate monitoring of diseases [102]. As a result, liquid biopsy provides more comprehensive information from a disease across multiple time points, enabling rapid and effective treatment.

Circulating tumor cells (CTCs) [101], exosomes [103], cell-free DNAs (cfDNAs) [104], and circulating microRNAs (miRNAs) [105] have emerged as potential biomarkers for monitoring human diseases. A number of studies have reported that the genomic or proteomic profiling of these biomarkers is associated with progression, proliferation, recurrence, chemo-sensitivity, and metastatic potential of the disease [106, 107]. However, accurate analysis and sensitive detection still remain a challenge due to the low concentration of liquid biopsy biomarkers in human bodily fluids [108]. Moreover, molecular heterogeneity among the biomarkers, coupled with phenotypic changes that frequently occur during therapeutic treatment and disease progression, makes separation of the biomarker difficult, limiting further downstream analysis [109].

This section summarizes several new technologies that use PNCs to detect liquid biopsy biomarkers with high sensitivity and specificity (Table 3). Antibodies are one of the most extensively used capture agents for separation of disease-related biomarkers, due to their high selectivity and strong binding affinity to specific surface receptors [102, 110]. Recent studies suggest that antibodies could be spliced into shorter peptides that still recognize specific surface receptors [111, 112]. As molecules that are small, stable, and easy to synthesize, compared to antibodies, peptides provide an opportunity to potentially replace the whole antibodies by addressing the reproducibility and productivity issues that current antibody-based approaches typically have [74]. Despite these advantages, the low binding affinity to specific target tissues are the major drawbacks of peptides. However, these concerns could be potentially addressed through the PNC approaches. For example, the multivalent binding effect, as described above, could be easily incorporated to various PNCs, which would improve biomarker separation based on the peptide binding to target biomarkers [37, 47].

**Table 3:** Peptide–nanoparticle conjugates for biomarker detection

Biomarker	Peptide (Pep)		Nanoparticle (NP)		In vitro studies		Clinical application	Refs.
	Target	Affinity (method)	Type	Size	In vitro model	Capture/detection		
CTC	EpCAM	K_D_: 2.69 × 10^−10^ M (SPR)	Iron oxide magnetic NP	235 nm (NP) 305 nm (conjugate)	MCF-7, SK-BR-3 (breast), PC3 (prostate), Hep G2 (Liver)	90% (capture) 93% (purity) > 90% (viability)	N/A	[111]
	HER2	Capacity: 70% Selectivity: 0.7, (FCM, compared to anti-HER2 Ab)	Iron oxide magnetic NP	200 nm	MCF-7, SK-BR3 (Breast), SKOV3 (ovarian)	75% (capture)	N/A	[112]
	EGFR	K_D_: 4.59 × 10^−4^ M (AFM)	Magnetic nanovesicles	219 nm	SMMC-7721 (hepatoma)	90% (capture)	Tested with 25 lung cancer patients’ samples.	[114]
	EGFR	N/A	AuNP	60 nm	Tu212 (head and neck), H292, H460 (Lung), MDA-MB-231 (Breast)	1–720 CTCs/mL (sensitivity) > 10^4^:1 (specificity) * SERS detection	Tested with 19 head and neck cancer patients’ samples	[115]
Exosome and extracellular Vesicle	CD63	N/A	Nickel Dynabeads	N/A	Human serum obtained from 10 healthy volunteers	54% (capture)	Tested with 15 HCC and 18 pancreatic patients’ samples	[118]
	Hsp70	N/A	Streptavidin-coupled Dynabeads	N/A	Lysates from MCF-7 (breast)	Similar with ultracentrifugation (capture)	N/A	[121]
cfDNA and miRNA	miR-21, miR-96, miR-125b	N/A	Nano metal—organic framework (NMOF, UiO-66)	125 nm	Synthetic miRNA MCF-7, MDA-MB-231 (breast), MCF-10A (non-tumor, breast)	< 10 pM (limit of detection)	N/A	[124]
	miR-21, miR-96, miR-125b	N/A	Nanosized graphene oxide	0.05–300 nm (lateral) 1.03 nm (height)	Synthetic miRNA MCF-7, MDA-MB-231 (breast), MDA-MB-435 (melanoma), HeLa (Cervix)	1 pM (limit of detection)	N/A	[125]
	let-7b, let-7c, miR-21	N/A	AuNP	10 nm	Synthetic miRNA + human serum	< 10 fM (limit of detection) one-base mismatch (selectivity)	N/A	[126]
	E542K, E545K, methylation of PIK3CA gene	N/A	AuNP	50 nm	Synthetic ctDNA + human serum	50 fM (limit of detection)	N/A	[127]

The Yang and Wang groups utilized peptides that recognize epithelial cell adhesion molecule (EpCAM) and human epidermal growth factor receptor 2 (HER2) for CTC isolation [111, 112]. These peptides were conjugated to iron oxide magnetic NPs for immunomagnetic separation. Although peptides themselves displayed lower binding affinity relative to antibodies, the PNC-based approach demonstrated over 90% and 70–80% of EpCAM-positive and HER2-positive cancer cell capture efficiencies, respectively, likely due to multivalent interactions. The epidermal growth factor receptor (EGFR) is another well-recognized tumor-specific antigen capable of targeting EpCAM-negative CTCs [113]. Ding et al. prepared nanovesicles with EGFR-targeting GE11 peptides distributed on their bilayers and magnetic NPs embedded into the vesicles using reverse phase evaporation [114]. The EGFR peptide magnetic vesicles (EPMVs) were able to bind to a hepatoma cancer cell line, SMMC-7221, showing a capture yield of 90%. EPMVs subsequently showed significant improvement in CTC isolation from the blood of lung cancer patients, outperforming both the CellSearch system and EpCAM-based immunomagnetic separation. The EGFR peptides were also conjugated with surface-enhanced Raman scattering (SERS) AuNPs to identify and characterize CTCs [115]. The in vitro results indicated over 90% cancer cell capture efficiency and 10^4^:1 detection specificity. Further clinical pilot studies revealed that the EGFR-specific PNCs detected up to 720 CTCs/mL from head and neck cancer patients’ samples.

Exosomes are endosomally derived extracellular vesicles (EVs) that play a major role in in intercellular signaling [103, 116]. It has been well established that exosomes carry proteins and genomic information of their parental cells [117]. Thus, great efforts have been made to isolate tumor-associated exosomes from various EVs in human bodily fluid. Tetraspanin CD63, a surface protein overexpressed in human exosomes, has been widely used to capture these vesicles [103]. Gao et al. recently reported a novel NP that has CD63-targeting peptides coated on its surface [118]. This exosome-targeting NP achieved a 54% capture rate when compared to the ultracentrifugation method. Clinical trials using human serum samples have demonstrated overexpression of tumor-related proteins, AFP and GPC-1, on the captured EVs, which are well-defined indicators of hepatic and pancreatic tumor, respectively. Other tumor-specific receptors have also been targeted to identify EVs secreted from tumors. Heat shock protein 70 (Hsp70), which acts as molecular chaperone, is highly expressed on majority of tumor cells [119, 120]. Ghosh et al. employed Vn96, a Hsp70-specific peptide, to isolate EVs derived from cancerous cells [121]. Vn96 peptides were densely coated on nanospheres and incubated with lysates obtained from MCF-7, a Hsp70-positive cancer cell line. The peptide–NP conjugates successfully isolated Hsp70-presenting EVs from human serum, showing comparable capture efficiency to ultracentrifugation.

Circulating nucleic acids are another biomarker of interest, encompassing cfDNAs and miRNAs. The utility of circulating nucleic acids (NAs) has been investigated for several decades because the NA fragments that are released from the tumor may possess the entire genomic information of heterogeneous tumor cells [122]. Peptide nucleic acids (PNAs) have recently been utilized by numerous research groups for detecting specific mutations in circulating NAs. PNAs are artificially synthesized NA analogues, that have increased long-term stability and enhanced binding with complementary sequences compared to natural NAs [123]. Combinations of PNA probes with NPs enable sensitive and selective quantification of circulating NAs. PNA probes have been coupled with various NPs, including nano metal–organic frameworks (NMOFs) [124], nano-sized graphene oxides (NGOs) [125], or AuNPs [126, 127], depending on how they quantify NAs. The most well-established approach measures changes in fluorescent signals. For example, tight binding between NMOF or NGO with PNA probes results in fluorescence quenching, which can be recovered when PNA probes are released from the complex via hybridization with specific miRNA [124, 125]. Using this methodology, both NMOF- and NGO-conjugated NPs can successfully detect targeted miRNAs, even at concentrations below 10 pM. AuNPs are also frequently conjugated with PNA probes. miRNA or ctDNA adsorption on the surface of PNA–AuNP conjugates subsequently alters the electrical, optical, and plasmonic properties of the conjugates. Nguyen et al. applied peptides conjugated to AuNPs for the detection of tumor-specific mutations E542K, E545K, and methylation of PIK3CA gene [127]. Adsorption of ctDNA onto PNA–AuNP conjugates shifted the localized surface plasmon resonance (LSPR) peak from 4.3 to 11.4 nm, showing 107% LSPR peak-shift compared to the primary response. This novel strategy allowed the detection of ctDNAs down to 50 fM.

Despite lower binding affinity of free peptides, multivalent binding effect of the PNCs allows these short chain amino acid compounds to be utilized as capture agents for liquid biopsy with comparable capture efficiency to the devices using antibodies. However, the majority of PNC-based liquid biopsy platforms are still in the early stage of development; only a limited number of such devices have demonstrated clinical utilities. Further downstream analysis of the captured biomarkers, including molecular characterization and functional assays, would potentially enhance clinical applicability of the PNC-based liquid biopsy platforms.

## Summary and outlook

Molecularly poised between proteins and small molecular compounds, peptides can potentially exploit structural and functional advantages of the two major materials in pharmacological research. As summarized above, a number of peptides, combined with NPs, have shown that their promising potential in the area of drug delivery, inhibition of pathogenic biomolecular interactions, molecular imaging, and liquid biopsy. Despite the potential, clinical translation of PNCs still remains elusive due to the following reasons. First, the PNC behaviors in physiological conditions, such as bloodstream and intracellular space, have not been fully understood. Second, peptides are still vulnerable to enzymatic degradation even on nanomaterial surfaces [128], requiring additional protection strategies to maintain their functions without increasing the structural and compositional complexity of the conjugates. Third, the potential immunogenicity of the engineered PNCs should be addressed, which is a common obstacle for in vivo and clinical application [129]. Lastly, covalent conjugation with NPs or other functional moieties often results in the loss of the biological functions of the peptides.

Upon addressing those concerns, it is certain that the PNC systems would provide a novel class of materials that potentially fill the gap in current biomedical areas, such as drugging ‘undruggable’ targets, combating against multidrug resistant pathogens, isolating rare biomarkers from human body fluids, and utilizing as submicron-molecular imaging agents. Particularly along with the rapid advances in nanotechnology, the PNCs will likely become a new platform that can be used in mainstream therapeutic and diagnostic systems.
